# Triceps Surae Muscle-Tendon Unit Properties in Preadolescent Children: A Comparison of Artistic Gymnastic Athletes and Non-athletes

**DOI:** 10.3389/fphys.2019.00615

**Published:** 2019-05-21

**Authors:** Nikolaos Pentidis, Falk Mersmann, Sebastian Bohm, Erasmia Giannakou, Nickos Aggelousis, Adamantios Arampatzis

**Affiliations:** ^1^Department of Training and Movement Sciences, Humboldt-Universität zu Berlin, Berlin, Germany; ^2^Berlin School of Movement Science, Humboldt-Universität zu Berlin, Berlin, Germany; ^3^Department of Physical Education and Sport Science, Democritus University of Thrace, Komotini, Greece

**Keywords:** preadolescent athletes, tendon stiffness, muscle strength, jumping performance, training documentation

## Abstract

Knowledge regarding the effects of athletic training on the properties of muscle and tendon in preadolescent children is scarce. The current study compared Achilles tendon stiffness, plantar flexor muscle strength and vertical jumping performance of preadolescent athletes and non-athletes to provide insight into the potential effects of systematic athletic training. Twenty-one preadolescent artistic gymnastic athletes (9.2 ± 1.6 years, 15 girls) and 11 similar-aged non-athlete controls (9.0 ± 1.7 years, 6 girls) participated in the study. The training intensity and volume of the athletes was documented for the last 6 months before the measurements. Subsequently, vertical ground reaction forces were measured with a force plate to assess jumping performance during squat (SJ) and countermovement jumps (CMJ) in both groups. Muscle strength of the plantar flexor muscles and Achilles tendon stiffness were examined using ultrasound, electromyography, and dynamometry. The athletes trained 6 days per week with a total of 20 h of training per week. Athletes generated significantly greater plantar flexion moments normalized to body mass compared to non-athletes (1.75 ± 0.32 Nm/kg vs. 1.31 ± 0.33 Nm/kg; *p* = 0.001) and achieved a significantly greater jump height in both types of jumps (21.2 ± 3.62 cm vs. 14.9 ± 2.32 cm; *p* < 0.001 in SJ and 23.4 ± 4.1 cm vs. 16.4 ± 4.1 cm; *p* < 0.001 in CMJ). Achilles tendon stiffness did not show any statistically significant differences (*p* = 0.413) between athletes (116.3 ± 32.5 N/mm) and non-athletes (106.4 ± 32.8 N/mm). Athletes were more likely to reach strain magnitudes close to or higher than 8.5% strain compared to non-athletes (frequency: 24% vs. 9%) indicating an increased mechanical demand for the tendon. Although normalized muscle strength and jumping performance were greater in athletes, gymnastic-specific training in preadolescence did not cause a significant adaptation of Achilles tendon stiffness. The potential contribution of the high mechanical demand for the tendon to the increasing risk of tendon overuse call for the implementation of specific exercises in the athletic training of preadolescent athletes that increase tendon stiffness and support a balanced adaptation within the muscle-tendon unit.

## Introduction

Imbalances within muscle-tendon units affect the tendon safety factor [i.e., ratio of tendon ultimate strain to functional operating tendon strain (Ker et al., 1988)], because the ultimate strain of tendons cannot be significantly changed (LaCroix et al., 2013). Thus, in individuals with higher muscle strength, the margin of tolerated mechanical loading is commonly increased by means of greater stiffness of their tendons (Arampatzis et al., 2007b). A higher tendon stiffness will result in less tendon strain at a given tendon force, which might imply less damage, because the strain is the primary mechanical parameter governing tendon damage accumulation for both static and cyclic loading (Wren et al., 2003). However, the types of loading that trigger adaptation and the time course of adaptive changes are different between muscles and tendons, and therefore periods of imbalances in muscle and tendon properties can occur during training (Mersmann et al., 2016). The muscle has a higher metabolic rate compared with the tendon (Laitinen, 1967) and it is likely that muscles adapt at a higher rate to altered mechanical loading. For example, changes in the morphology and architecture of the muscle have been reported after 3 to 4 weeks of heavy resistance training (Seynnes et al., 2007; DeFreitas et al., 2011), while no such fast changes were observed in the mechanical or morphological tendon properties (Mersmann et al., 2017a). In adolescent athletes, it has been shown that an imbalance between muscle strength and tendon stiffness results in a higher mechanical demand for the tendon compared to non-athlete controls (Mersmann et al., 2017a).

Research of the last 15 years has shown that the mechanical properties of tendons are crucial for muscle-tendon unit functioning during walking (Lichtwark et al., 2007; Lai et al., 2015), running (Arampatzis et al., 2006; Albracht and Arampatzis, 2013; Lai et al., 2018), and jumping (Bojsen-Møller et al., 2005; Ishikawa and Komi, 2008; Nikolaidou et al., 2017). These studies have shown that the elasticity of tendons enables the storage and release of strain energy during movements, which facilitates the operating conditions of muscle fascicles with regard to the force-length-velocity relationships. This energy exchange within the muscle-tendon unit seems to be optimal at a given balance of muscle strength and tendon stiffness (Lichtwark et al., 2007; Orselli et al., 2017). Therefore, in healthy adults, muscle strength and tendon stiffness are usually strongly associated (Arampatzis et al., 2007b). Furthermore, an adequate strain applied to the tendon is important and necessary for tendon healthiness and adaptability. In previous studies (Arampatzis et al., 2007a, 2010; Bohm et al., 2014) we demonstrated that cyclic loading of the tendon, which caused tendon strain of approximately 4.5 to 6.5% for about 3 s per cycle, is a very effective stimulus to improve the mechanical, morphological and material properties of the tendon. Mechanical tendon loading that introduces low-level tendon strain (≤3%) on the other hand cannot improve tendon properties (Arampatzis et al., 2007a, 2010). However, tendon strain higher than 9% may overload the tissue, result in degeneration of the tendon and impair its structural integrity (Wang et al., 2013). Strain magnitudes higher than 8.5–9.0% during maximum isometric contractions might be indicative of imbalances within the muscle-tendon unit in terms of tendon stiffness being too low compared to muscle strength (Mersmann et al., 2016; Bohm et al., 2019).

Although training-induced muscle hypertrophy in preadolescent children is still debated (Ramsay et al., 1990; Ozmun et al., 1994; Malina, 2006; Granacher et al., 2011), resistance exercise and athletic training clearly improve muscle strength (Faigenbaum et al., 1999; Granacher et al., 2011; Behm et al., 2017) and jumping performance in preadolescence (Bassa et al., 2012; Marina and Memni, 2014; Moran et al., 2017). Yet, to our knowledge, no study has investigated the effect of athletic training on tendon mechanical properties in preadolescent children. A study by Waugh et al. (2014) found a significant increase in both muscle strength and tendon stiffness in prepubertal children after an exercise intervention of 10 weeks. However, the mechanical stimulus used in that study is known to be effective for tendon adaptation (i.e., high magnitude, 2–3 s duration strain) and might not be representative of the effects of sport-specific loading. In fact, certain types of sport-specific athletic training, at least in adolescent, seems to induce imbalances in the development of muscle strength and tendon stiffness increasing the mechanical demand for the tendon (Mersmann et al., 2016, 2017a). Knowledge about the effects of athletic training on the muscle-tendon properties in preadolescent children is important for the improvement of our understanding with respect to muscle and tendon plasticity in this age.

The main objective of the current study was to compare the Achilles tendon mechanical properties, plantar flexor muscle strength and vertical jumping performance of preadolescent athletes and non-athletes in order to estimate the potential effects of systematic athletic training. We hypothesized higher muscle strength, tendon stiffness and jumping performance in athletes compared to non-athlete controls.

## Materials and Methods

### Participants and Experimental Design

Twenty-one preadolescent artistic gymnastic athletes (6 males, 15 females; ≈20 h of sport-specific training per week) and a control group of 11 untrained participants (5 males, 6 females; ∼3 h of sport activity per week in school and no further sports participation) with an age of about 9 years (Table 3) agreed to participate in the study. The study was approved by the university ethics committee of the Democritus University of Thrace, and all participants (and respective legal guardians) signed informed consent to the experimental procedures. The pubertal status of each child was determined by their parents, and the children were classified according to pubic hair, genital and breast development as described by Marshall and Tanner (1969, 1970). Each parent determined the proper stage according to drawings representing the five different Tanner stages. Additionally, information on the skeletal age of the athletes was provided by the parents following a routine medical examination 1 month after our measurements. The diagnosis was done by a pediatric radiologist based on radiographic images obtained from the left hand of the participants. Due to the exposure to radiation, no radiographic images were made from the non-athletes group. The measurements of muscle strength and tendon mechanical properties were conducted on the right leg in every participant. Four females (2 athletes and 2 non-athletes) had a dominant left leg (i.e., leading leg in a forward fall). However, in participants who did not engage in physical activity related to strong unilateral loading, we did not expect significant differences in muscle- strength or tendon stiffness between sides (Bohm et al., 2015).

### Training Documentation

We started the training documentation 6 months before the measurements, in order to quantify the training intensity and training volume. The training was documented by one observer with experience in artistic gymnastics (N.P.) for 1 week (six training units) in the middle of each month (second or third week of the month). The training consisted of a warm-up, muscle strength exercises (mostly with body weight), specific exercises of the lower limbs for the improvement of the jump performance, which involved a variety of jumps and acrobatic elements, and finally the main training for each apparatus. All athletes performed the same number of repetitions (exercises) during warm-up, muscle strength and jump exercises. The only difference was present during the main training, as girls trained on one more apparatus for the lower limbs (i.e., balance beam). Based on our assessment, the variation of overall training volume between athletes was approximately ± 5%. A repetition was classified as medium intensity when during the execution the athletes focused more on a proper technique, and high intensity when the athlete had to perform the repetition with maximal effort. Detailed information regarding the training contents is presented in Table 1. Table 2 shows an example of a typical weekly training program including the number of repetitions and duration of the training contents.

**Table 1:** Detailed description of each exercise for the lower extremities documented for the quantification of the training intensity and volume.

Part of the training	Exercise	Description of the exercise
Warm-up	A-skip	Skipping forward, alternating lifting one knee to waist-height while keeping the other straight


	Butt kicks	Alternating flexing the knee and kicking the heel up toward the gluteus, bringing the foot back


	High knees	Lifting up the knee as high as it will go and raise the opposite arm, switching quickly so the left knee is up before the right foot lands


	Grapevine	Performing a criss-cross with the legs by stepping side-to-side


	Straight leg shuffle	Alternating moving the straight leg forward at high cadence


	Backward running	Pushing off the ground with the balls of the feet while moving backward


Muscle strength	Squat jump	1–2 kg ankle weights: Maximum jump following 1–2 s in a squat position


	Squat	10–20 kg barbell: Squats with 1–2 s with 1–2 s in squat position


	Calf raises	Plantar flexion using co-athlete (piggyback) as additional weight


	Plinth jump	Countermovement jump with arm swing onto and off a plinth of 45 to 70 cm height


	Vertical depth jump	Drop jump from the top of a plinth of 45 to 70 cm height


Jump performance improvement	Two-Legged hops	Repetitive two-legged long jumps (also called bunny hops)


	Power skipping	Fast skipping, lifting the upper leg as high as possible


	Alternate leg bounding	Running with long strides, placing emphasis on hang time


	Lateral jump	Lateral jump side to side (modification: with forward displacement)


	Tuck jump	Forward jump while flexing the hip and knees and in the air to a crouched position


	Pike jump	Forward jump with dynamic hip flexion in the air while keeping the legs straight


	Straddle jump	A vertical jump where the legs are lifted into an airborne straddle (90° wide open) with arms and trunk extended over the legs as they are elevated


Main training (apparatus)	Blockings	In gymnastics term “punch”: A rapid bounce off the floor or apparatus that converts horizontal speed to vertical


	Landings	Landings after jumps, acrobatic exercises and dismounts of the apparatus


	Jumps	Jumps appearing during the floor exercises and on the balance beam




**Table 2:** Description of the training documentation per week (six training units per week).

Part of the training	Sessions per week	Total repetitions^a^	Duration
Warm-up	6 / 6		180 min
Muscle strength	5 / 6	150 medium intensity	210 min
		450 high intensity	
Specific exercises for improvement of jump performance	5 / 6	225 medium intensity	75 min
		225 high intensity	
Main training [apparatus]	6 / 6	480 high intensity	720 min


*^*a*^Total repetitions: The sum of the plyometric exercises, jumps, blocking, and landing elements; high intensity was documented when the athlete was instructed to perform the exercise with maximal effort (e.g., jump as high/fast as possible); medium intensity was documented when the athlete was instructed to perform the exercise focusing more to technique rather than with maximal effort.*

### Measurement of Jump Performance

After a standardized warm-up including 3 min of running, 10 submaximal jumps and 5 submaximal isometric ankle plantar flexion contractions, each participant performed first three trials of maximum effort countermovement jumps (CMJ) and then three trials of squat jumps (SJ). The CMJ was performed starting from a standing position. The participants were instructed to quickly squat to a knee angle of approximately 90° (checked by visual observation by the investigator) and then to jump immediately as high as possible afterward. For the SJ, the participants were instructed to take the starting position with a knee angle of 90°, hold that position for 3 s, as determined by the experimenter, and then to perform the jump as high as possible without countermovement. In both jumps, the arms were held akimbo. The vertical ground reaction forces (GRF) during the jumps were measured with a force plate sampling at 1,000 Hz (Kistler 9281CA, Winterthur, Switzerland) and the vertical take-off velocity of the center of mass during the jumps was calculated through the integration of the vertical GRF over the time. Mechanical power applied to the center of mass was determined as the product of vertical ground reaction force and vertical center of mass velocity. The propulsion phase is defined in both SJ and CMJ from the start of the upward movement of the center of mass until take-off. The mean mechanical power was calculated by dividing the integral of power from the propulsion phase by propulsion time. Further, we calculated the eccentric utilization ratio (EUR) as the ratio of CMJ height (or mean mechanical power) to SJ height (or mean mechanical power). The trial with the greatest jump height was considered in the statistical analysis.

### Measurement of Maximum Ankle Joint Moment

Plantar flexor muscle strength was measured combining dynamometry, kinematic, and electromyography (EMG) recordings. Effects of gravitational forces and the misalignment of rotation axes of ankle joint and dynamometer were considered using inverse dynamics (Arampatzis et al., 2005). For this purpose, seven reflective markers were fixed to the following anatomical landmarks: trochanter major, the most prominent points of the lateral and medial femoral epicondyles, lateral and medial malleolus, the dorsal gap between the distal metaphysis of the 2nd and 3rd metatarsals and tuber calcaneus, as well as five markers fixed on the dynamometer: axis of the dynamometer, lever of the dynamometer, two markers on the foot plate located lateral and medial to the line of force application and one additional to define the surface of the foot plate. Kinematic data were recorded using a Vicon motion capture system (version 1.8.5; Vicon Motion Systems, Oxford, United Kingdom) integrating eight cameras operating at 100 Hz. The electromyographic (EMG) activity of the head of the tibialis anterior was recorded using two bipolar surface electrodes (Blue Sensor N, Ambu GmbH, Bad Nauheim, Germany) fixed over the mid-portion of the muscle belly with an inter-electrode distance of 2 cm after shaving and cleaning the skin. EMG data was captured at 1,000 Hz (Myon m320RX; Myon, Baar, Switzerland) and transmitted to the Vicon system via a 64-channel A-D converter. The participants were asked to perform five isometric maximum voluntary ankle plantar flexion contractions (MVC) on a dynamometer (Cybex 6000, Ronkonkoma, New York, NY, United States) at 70° trunk flexion (supine = 0°) with the knee fully extended (180°) and the ankle joint at 0°, 5°, 10°, 15°, and 20° dorsiflexion (0° = tibia perpendicular to the sole), respectively. If a participant was not able to reach some of these degrees of dorsiflexion, those trials were not performed. All the athletes were able to perform the above mentioned five trials, while the non-athletes were able to reach a maximum of 15° dorsiflexion. A passive ankle plantar- and dorsiflexion trial (driven by the dynamometer at 5°/s) and two trials of dorsiflexion contractions were recorded to account for gravitational forces and to establish an activation-flexion moment relationship that was used to estimate the ankle dorsiflexion moment generated during maximum effort ankle plantar flexion due to antagonistic co-activation (Mademli et al., 2004).

### Measurement of Tendon Mechanical Properties

For the assessment of the Achilles tendon mechanical properties, the participants completed five trials of isometric ramp contractions (i.e., increasing effort steadily from rest to maximum in ≈4 s followed by a plateau at maximum effort of 2–3 s) with the ankle joint angle at 0° and rest between ramp contractions of 3 min. The displacement of the myotendinous junction (MTJ) of the gastrocnemius medialis muscle-tendon unit during the contractions was captured at 85 Hz by a 60 mm linear ultrasound probe (7.5 MHz; Chison, Model Q3, Wuxi, China). The ankle plantar flexion moments were calculated using the same approach as for the MVC calculation (i.e., correction for axes misalignments, gravitational forces, and antagonistic co-activation), and the ultrasound images were synchronized offline with the data captured in the Vicon system using an externally induced voltage peak, which could be identified in both the ultrasound and the analog data stream. The effect of unavoidable joint angular rotation on the displacement of MTJ during the ramp contractions was taken into consideration by capturing the displacement of the MTJ during a passive rotation of the ankle joint (Arampatzis et al., 2008). The elongation of the tendon was calculated as the difference of the MTJ displacement measured during the active contractions and the passive joint rotation. To measure the resting length of the Achilles tendon, the participants were seated on the dynamometer with fully extended knee and the ankle at -20° (plantar flexion), as in this position the inactive gastrocnemius medialis muscle-tendon unit is slack (De Monte et al., 2006). The length of the curved path from the calcaneal tuberosity to the MTJ was defined as the resting length.

Tendon force was calculated by dividing the ankle plantar flexion moment by the tendon moment arm, which was calculated based on the approach suggested by Fath et al. (2010). In short, the moment arm was estimated based on the relationship of MTJ displacement and ankle angular rotation between 5° dorsiflexion and -10° plantar flexion [i.e., range of negligible passive tendon strain; De Monte et al. (2006)]. The moment arm was adjusted to changes induced by force application based on the data of Maganaris et al. (2000). The displacement of the MTJ during the ramp contractions and passive trials was digitized manually by tracking its position frame by frame using a custom-written MATLAB interface (The MathWorks, version 2012b, United States). The tracking was done by one experienced observer (N.P.) and the force-elongation relationship of the five trials of each participant was averaged to achieve excellent reliability (Schulze et al., 2012). Tendon stiffness was then calculated between 50 and 100% of the peak tendon force of the averaged force-elongation curve. We further calculated the normalized tendon stiffness (i.e., the product of stiffness and rest length) that represents the slope of the force-strain curve.

### Statistics

The statistical analyses were conducted in SPSS (version 25.0; IBM, Armonk, NY, United States). Normal distribution of the data was checked for all parameters using the Shapiro-Wilk test. For the non-normally distributed parameters, which were body mass, body mass index, co-activation, and height of the CMJ, we compared the groups using the non-parametric Krustal–Wallis one-way analysis of variance for independent samples. For all other parameters we performed a one-way analysis of covariance (ANCOVA) for the effect of group (i.e., athletes and non-athletes) with sex as covariate. We calculated the effect size (*f*) for statistically significant observations in G^∗^Power (Version 3.1.6; HHU, Düsseldorf, Germany; Faul et al., 2007) based on the partial eta squared. The level of significance for all comparisons was set to α = 0.05. In all figures, the data are presented as mean ± standard error of mean (SEM), whereas in the text and tables they are expressed as mean ± standard deviation (SD).

## Results

There were no significant differences of between the two groups with regard to age (*p* = 0.906), body height (*p* = 0.339), mass (*p* = 0.812), body mass index (B.M.I.) (*p* = 0.984), and tibia length (*p* = 0.394; Table 3). The majority of the athletes were in Tanner stage I, while more non-athletes were in stage II than in I (Table 3). According to the evaluation of the radiographic images of the athletes, their skeletal age was on average 1 year less (1 ± 0.8 yr) compared to the chronological age. The athletes completed a training program of about 20 h per week (over 6 days per week). Based on our assessment regarding the intensity of the training (i.e., low intensity during warm-up, medium to high intensity during the muscle strength and the gymnastic specific training, and high intensity during the main on-apparatus training), we found almost an equal intensity distribution among the training (34% low, 30% medium, and 36% high).

**Table 3:** Anthropometric data and Tanner scale of the preadolescent athletes and non-athletes.

	Athletes (*n* = 21)	Non-athletes (*n* = 11)
Age [years]	9.2 ± 1.7	9.0 ± 1.7
Height [cm]	131.1 ± 8.0	134.6 ± 11.7
Body mass [kg]	28.9 ± 6.4	31.1 ± 9.0
Tibia length [cm]	28.7 ± 2.2	29.4 ± 2.7
BMI [kg/m^2^]	16.6 ± 2.0	16.9 ± 3.0
Percentage in Tanner stage I	66.6%	45.5%
Percentage in Tanner stage II	33.4%	54.5%


*Values are means ± standard deviation (SD). BMI: Body mass index. There were no significant differences between groups in the anthropometric data. Values for the Tanner scale are in percentage of the total participants in each group.*

**FIGURE 1:**
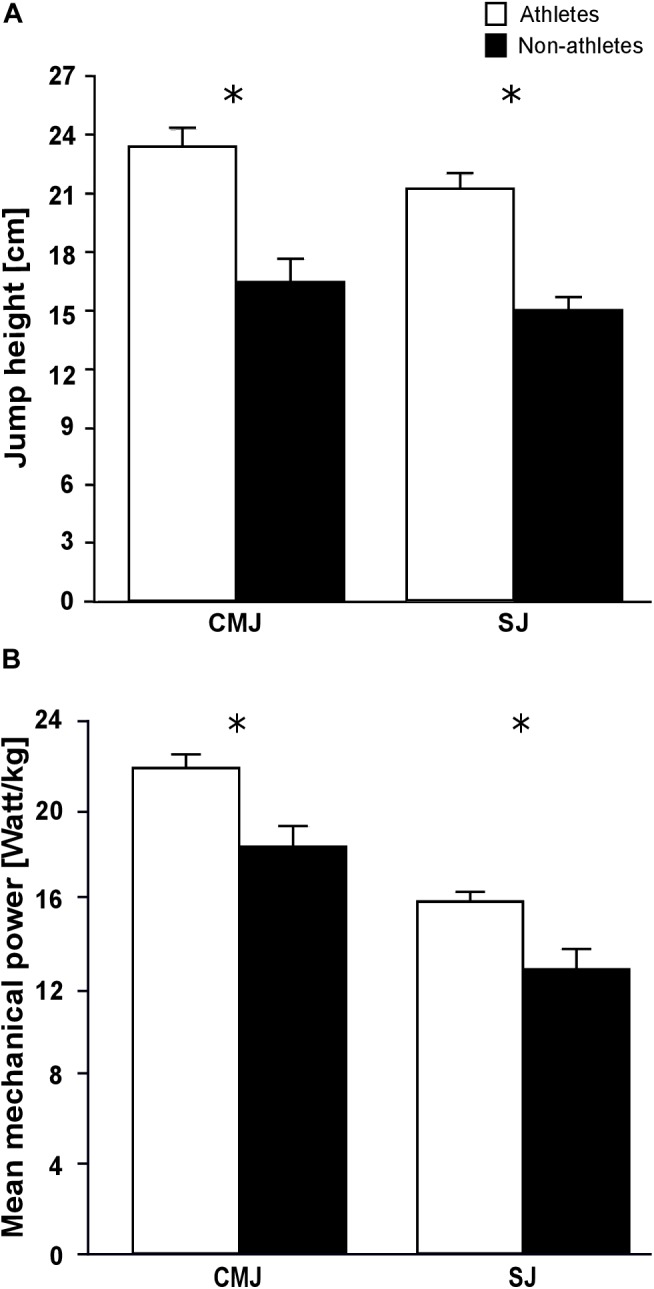
Mean values and standard error (error bars) of **(A)** jump height and **(B)** mean mechanical power during the propulsion phase of preadolescent athletes and non-athletes during the countermovement (CMJ) and squat jump (SJ). ^∗^Significant difference between athletes and non-athletes *p* < 0.05.

The maximum plantar flexion moments normalized to body mass were significantly greater in the athletes compared to the non-athletes (*f* = 0.65, *p* = 0.001; Table 4). However, the non-normalized maximum plantar flexion moments did not show statistically significant differences between athletes and non-athletes (*p* = 0.06; Table 4). Athletes showed a significantly lower co-activation than the non-athletes during the maximum plantar flexion contraction (*f* = 0.45, *p* = 0.021; Table 4). We found significantly greater jump height in athletes compared to non-athletes in both jumps (*f_SJ_* = 0.94, *f_CMJ_* = 0.83, *p* < 0.001 for the SJ and CMJ, respectively; Figure 1A), as well as greater mean mechanical power during the propulsion phase (*f* = 0.6, *p* = 0.004 for the SJ and CMJ; Figure 1B). The duration of the propulsion phase did not show any significant differences between the two groups in both SJ (*p* = 0.132) and CMJ (*p* = 0.251; Table 4). The EUR_height_ and EUR_power_ also did not show any significant differences between groups (*p* = 0.852 and *p* = 0.209, respectively; Table 4).

**Table 4:** Ankle joint moment and jump performance of the preadolescent athletes and non-athletes.

	Athletes	Non-athletes
	(*n* = 21)	(*n* = 11)
Maximum plantar flexion moment [Nm]	50.6 ± 14.6	40.4 ± 14.1
Normalized max. plantar flex. moment [Nm/kg]^∗^	1.75 ± 0.32	1.31 ± 0.33
Antagonistic co-activation [%]^∗^	4.0 ± 2.2	7.1 ± 4.5
Propulsion time during SJ [ms]	316 ± 42	352 ± 71
Propulsion time during CMJ [ms]	260 ± 37	247 ± 61
Eccentric utilization ratio [jump height]	1.10 ± 0.12	1.09 ± 0.19
Eccentric utilization ratio [mean mechanical power/kg]	1.39 ± 0.18	1.50 ± 0.35


*Values are means ± standard deviation. Maximum plantar flexion moment was normalized to body mass. Antagonistic co-activation is the antagonistic moment normalized to the resultant ankle joint moment. SJ: Squat jump, CMJ: Countermovement jump. ^∗^Significant difference between athletes and non-athletes, *p* < 0.05.*

**FIGURE 2:**
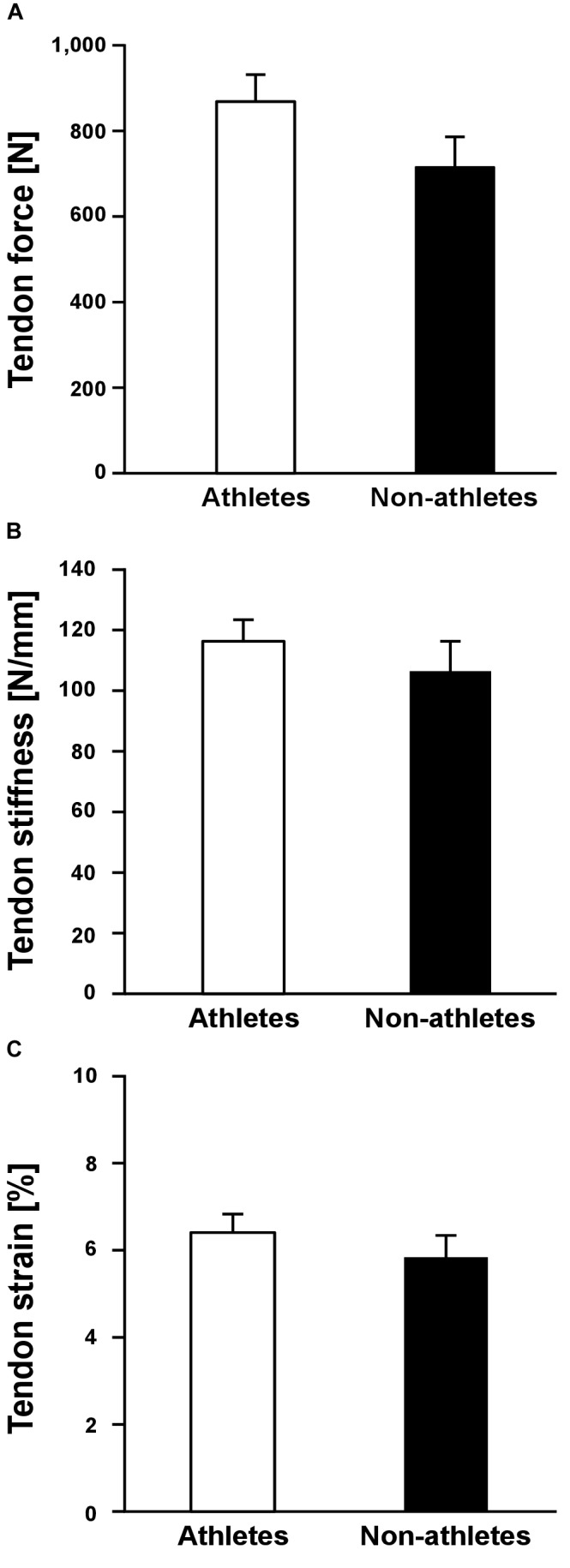
Mean values and standard error (error bars) of Achilles tendon force **(A)**, tendon stiffness **(B)** and tendon strain **(C)** during maximum isometric contractions of preadolescent athletes and non-athletes. No significant differences (*p* > 0.05) were found between the two groups.

**FIGURE 3:**
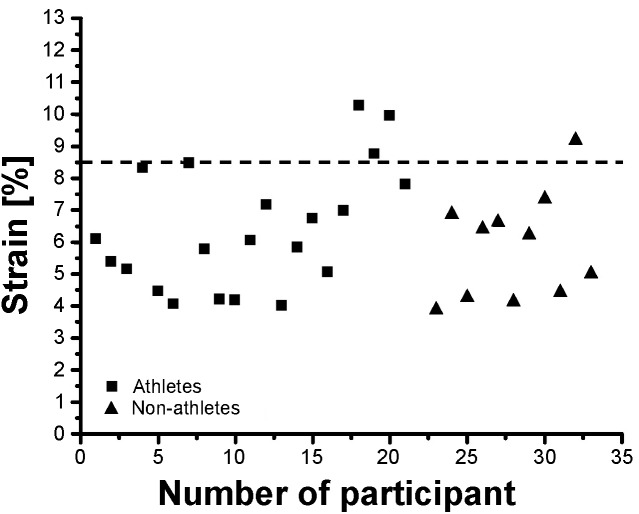
Individual Achilles tendon strain values during maximum isometric contractions in the investigated preadolescent athletes and non-athletes.

Athletes and non-athletes did not show any significant differences in maximum Achilles tendon force (*p* = 0.086; Figure 2A), Achilles tendon stiffness (*p* = 0.413; Figure 2B), normalized Achilles tendon stiffness (*p* = 0.513; Table 5) and maximum Achilles tendon strain (*p* = 0.222; Figure 2C). Finally, we did not find any statistically significant differences between the two groups in the Achilles tendon moment arm (*p* = 0.587; Table 5), rest length (*p* = 0.707; Table 5) and maximum tendon elongation (*p* = 0.152; Table 5). However, when examining the individual tendon strain values during the maximum isometric contractions, it is notable that athletes were more likely to reach strain magnitudes close to or higher than 8.5% strain compared to non-athletes (frequency of 24% in athletes and 9% in non-athletes; Figure 3).

**Table 5:** Achilles tendon properties of the preadolescent athletes and non-athletes.

	Athletes (*n* = 21)	Non-athletes (*n* = 11)
Normalized Achilles tendon stiffness [kN/strain]	14.5 ± 5.6	13.1 ± 5.7
Moment arm [mm]	42.2 ± 6.0	40.7 ± 9.1
Rest length [mm]	128.8 ± 22.3	124.3 ± 25.3
Maximum elongation [mm]	7.71 ± 2.1	6.85 ± 1.6


*Values are means ± standard deviation. Achilles tendon stiffness was normalized to tendon rest length.*

## Discussion

The present study compared the Achilles tendon mechanical properties, plantar flexor muscle strength and vertical jumping performance of preadolescent athletes and non-athletes to estimate the potential effects of systematic athletic training. We hypothesized higher values in all of these parameters in athletes compared to an age-matched group of non-athletes. We found greater jumping performance and muscle strength normalized to body mass in athletes, but no differences in tendon stiffness between the two groups. Therefore, our results only partly confirmed our hypothesis.

The investigated athletes completed a training program of about 20 h per week (over 6 days per week) with almost 5 h of specific muscle strength and jumping training. Further, the main on-apparatus gymnastic training of about 12 h included several plyometric and landing exercises. These data show that the training volume in the athletes was quite high and could account for an increase in muscle strength. From different studies in the previous years (Faigenbaum et al., 1996; Falk and Eliakim, 2003; Granacher et al., 2011, 2016) it is well accepted that even in childhood, physical exercise (e.g., resistance training, plyometric training) can increase muscle strength. There are several studies (e.g., Faigenbaum et al., 1999; Chaouachi et al., 2014) and reviews (Malina, 2006), which reported increases in muscle strength following plyometric, balance and resistance training in prepubertal children, with strength gains of up to 40% after 8 weeks (Faigenbaum et al., 1999). Also, two meta-analyses of Payne et al. (1997) and Behringer et al. (2010) calculated effect sizes of 0.75 and 1.12, respectively, providing evidence that resistance training increases muscle strength in children. Therefore, our findings of greater normalized ankle plantar flexor strength in athletes compared to non-athletes were not surprising. Considering that non-athletes were only slightly and not statistically significantly heavier compared to the athletes, we expected greater absolute ankle plantar flexor moments for athletes. Surprisingly, the maximum absolute ankle joint moments did not differ significantly between athletes and non-athletes. One explanation could be the lower state of maturity of the athletes, indicated by the higher percentage of athletes in the first Tanner stage than in non-athletes. Further, the radiographic images provided evidence that the athletes were delayed about 1 year in their skeletal development compared to their chronological age. Although the chronological age was similar in the two groups, the state of maturation seemed to be decelerated in the athletes, which is likely to affect the muscle strength development as well. In fact, when accounting for the difference in the maturation status by including the Tanner stage as additional covariate in the ANCOVA, we found significant differences (*p* = 0.001) in maximum plantar flexor moment between the two groups. Therefore, the missing differences in absolute muscle strength were very likely due to this difference in maturation. Several studies in the past reported a delay of one to 3 years of the skeletal compared to the chronological age in early- and mid-adolescent gymnasts (Novotny and Taftlova, 1971; Beunen et al., 1981; Claessens et al., 1991; Georgopoulos et al., 2004). Irrespective of the generalizability of these findings (see Malina et al., 2013) or the attribution of this delay to mechanical loading, energy balance (due to caloric restriction), selection or a concert of factors (Baxter-Jones et al., 1994; Rogol et al., 2000), which were beyond the scope of our study, our data suggest that a delay in the maturation process can occur in athletic gymnasts even before puberty.

Achilles tendon stiffness did not differ significantly between the two groups, despite the fact that the exercise loading was essentially higher in athletes. In adolescent athletes, Mersmann et al. (2017a) found volleyball athletes have both greater patellar tendon force and stiffness compared to an age-matched control group of non-athletes. Compared to the findings of the current study this indicates that the effects of athletic training on the adaptation of the muscle-tendon properties in preadolescent children and adolescents might be different. Differences in the hormone status between preadolescents and adolescents (Korth-Schutz et al., 1976; Round et al., 1999; Veldhuis et al., 2000) could provide some explanation for the different effects of training. The concentration of testosterone and oestrogen levels in preadolescence (i.e., Tanner stage I and II) is negligible (Round et al., 1999). However, these concentrations increase significantly after reaching Tanner stage III (Viru et al., 1998, 1999), which promotes the development and increase of muscle mass, physiological muscle cross-sectional area (Richmond and Rogol, 2007; Vingren et al., 2010) and the anabolic response of muscle to training (Hansen and Kjaer, 2014). It is well accepted that maximum muscle strength, or more precisely the force applied to the tendon during maximum isometric contractions, associates to tendon stiffness (Arampatzis et al., 2007b; Seynnes et al., 2009) and is an important mediator for tendon adaptation in both adults (Seynnes et al., 2009) and during childhood development (Waugh et al., 2012). Thus, it seems likely that the absence of marked differences in tendon force between athletes and non-athletes was the reason for the similar Achilles tendon stiffness. The clear differences in tendon force that were present between the respective late-adolescent cohorts in our earlier work (Mersmann et al., 2017a) explain the different results between studies. This lends support to the idea that hormonal changes during puberty promote the responsiveness of muscle to mechanical loading, especially with regard to muscle hypertrophy, which leads to more apparent differences in both muscle strength and tendon stiffness between athletes and untrained counterparts.

The tendon strain during the maximum isometric contractions was not significantly different between athletes and non-athletes in the present study, suggesting that athletic training in preadolescence does not increase the mechanical demand of the tendon as in adolescence (Mersmann et al., 2016), despite comparable mechanical loading during the training process. However, looking at the individual strain values during the maximum isometric contractions, it is notable that athletes were more likely to reach high strain magnitudes, which would indicate a high mechanical demand for the tendon and might indicate imbalances within the muscle-tendon unit. Further, when accounting for the apparent differences in maturation by including the Tanner stage as covariate in the statistical analysis, the differences in tendon stiffness were, in contrast to muscle strength, not significantly different between groups (*p* = 0.364). This also suggests that the athletic activity of the artistic gymnasts might lead to a stronger and/or more consistent adaptation in the plantar flexor muscles compared to its tendon. Thus, although the average tendon strain did not differ between the two groups, at least in some preadolescent athletes a specific training to increase Achilles tendon stiffness is recommendable from a preventive point of view to reduce the mechanical demand for the tendon. Waugh et al. (2014) reported an increase of Achilles tendon stiffness after 10 weeks of strength training in prepubertal children, indicating a possible adaptive potential of tendon mechanical properties in preadolescence. The specific athletic training for the lower extremities in our athletes was dominated by jumping, landing and plyometric exercises, which are the specific loading characteristic in artistic gymnastics. This kind of loading is characterized by high load magnitude and short duration of loading, which results in a high strain rate in the Achilles tendon. Several studies reported a low potential of plyometric training on tendon adaptation despite the high magnitude of tendon loading (Burgess et al., 2007; Kubo et al., 2007; Fouré et al., 2009, 2011; Bohm et al., 2014). Though this is not a consistent finding (Burgess et al., 2007; Wu et al., 2010; Hirayama et al., 2017), all studies directly comparing the effects of plyometric training to training routines that used lower strain rates and longer strain durations consistently show lower adaptive responses in the plyometric training groups (Burgess et al., 2007; Kubo et al., 2007; Bohm et al., 2014). Systematic research of our group provided evidence that an effective training stimulus for tendon adaptation is a combination of high loading magnitude, an appropriate load duration in every repetition (i.e., 3 s) and repetitive loading (Arampatzis et al., 2007a, 2010; Bohm et al., 2014). It can be argued that in some athletes in the present study, the sport-specific loading triggered an increase of muscle strength without sufficiently stimulating the adaptation of the tendon, which resulted in high mechanical demand for the tendinous structures. Therefore, specific tendon training to increase tendon stiffness is, at least in some individuals, recommended also in preadolescent athletes.

In both athletes and non-athletes, the jump height in the CMJ was 10% greater compared to the SJ and the mean mechanical power during the propulsion phase was 38% greater in the athletes and 43% in the non-athletes. Both groups appeared to utilize the stretch-shortening cycle equally to augment jumping performance. Comparable differences in jumping height and mean mechanical power between SJ and CMJ have been reported in adult athletes and non-athletes (Bobbert et al., 1996; Pazin et al., 2013; Nikolaidou et al., 2017), which indicates similar neuromuscular mechanisms for promoting jump height using a countermovement in preadolescent children. The jumping height in both SJ and CMJ was 30% greater in athletes and the mean mechanical power during the propulsion phase was 20% and 16% greater in SJ and CMJ, respectively, compared to the non-athlete controls. Although the contribution of the plantar flexors to the jumping height and mean mechanical power during the propulsion phase is important [i.e., 29 and 13%, respectively, (Nikolaidou et al., 2017)] other muscles within the lower extremities (i.e., knee and hip extensors) also contribute to the jumping performance (Anderson and Pandy, 1993; Nikolaidou et al., 2017). Exercises as for example hopping, lateral, pike and straddle jumps, blocking, and landings were substantial components of the training in the investigated athletes. In average the athletes participating in our study performed on a weekly basis approximately 1,300 repetitions of jumps either with medium or high intensity (medium focusing to proper technique and high with maximal effort), which indicates an increased mechanical load of all muscles within the lower extremities compared to the non-athlete control group. Furthermore, this specific gymnastic training with the large amount of plyometric loading can improve the neuromuscular coordination between the lower extremity muscles (Komi, 2000; Markovic and Mikulic, 2010; Ache-Dias et al., 2016) enhancing the performance during SJ and CMJ. It is well documented that plyometric training improves the mechanical power output and height during vertical jumping (Brown et al., 1986; Adams et al., 1992; Toumi et al., 2001) and, therefore, the specific gymnastic training can explain the better jumping performance of our athletes.

## Conclusion

In conclusion, the present study demonstrated that artistic gymnastic training during preadolescence is associated with increased plantar flexor strength normalized to body mass and jumping performance, without significant effects on Achilles tendon stiffness and absolute muscle strength compared to non-athletes. The delayed maturation of the athletes and the specific training load of artistic gymnastics are likely to explain these findings. In some individual athletes, an increased level of tendon strain during maximum isometric contractions indicates imbalances between muscle strength and tendon stiffness, resulting in a high mechanical demand for the tendon. The potential contribution of the increased mechanical demand to the high risk of tendon overuse in the athletic population call for the implementation of specific exercises into the athletic training of preadolescent athletes that effectively increase tendon stiffness and support a balanced adaptation within the muscle-tendon unit. To increase tendon stiffness, we recommend exercises that combine high intensity, repetitive loading and a long duration of loading (of ≈3 s) in every repetition (for review and exemplary exercises see Mersmann et al., 2017b). Finally, with regard to the inherent limitations of cross-sectional studies, which cannot rule out genetic influences biasing the inferences made regarding the effect of training, future research should aim to investigate the effects of athletic training during preadolescence using a longitudinal design.

## Ethics Statement

The study has been approved by the university ethics committee of Democritus University of Thrace, and all participants (including legal guardians) signed informed consent to the experimental procedures in accordance with the Declaration of Helsinki.

## Author Contributions

NP and AA conceived the experiments. NP and EG performed the experiments. NP analyzed the data. FM, SB, and AA substantially contributed to data analysis. NP, FM, and AA interpreted the data. NP and AA drafted the manuscript. FM, SB, EG, and NA made important intellectual contributions during revision. All authors approved the final version of the manuscript and agreed to be accountable for the content of the work.

## Conflict of Interest Statement

The authors declare that the research was conducted in the absence of any commercial or financial relationships that could be construed as a potential conflict of interest.
